# Functional Microbiomes at the Interface: Mediators in Marine Biofouling and Larval Settlement

**DOI:** 10.3390/ijms27104155

**Published:** 2026-05-07

**Authors:** Sergey Dobretsov, Daniel Rittschof, Lihua Peng, Jin-Long Yang

**Affiliations:** 1 Department of Marine Science and Fisheries, College of Agricultural and Marine Sciences, Sultan Qaboos University, Al-Khoud, P.O. Box 34, Muscat 123, Oman; 2 UNESCO Chair in Marine Biotechnology, Center of Excellence in Marine Biotechnology, Sultan Qaboos University, Al-Khoud, P.O. Box 50, Muscat 123, Oman; 3 Nicholas School of the Environment, Duke University, 135 Duke Marine Lab Road, Beaufort, NC 28516, USA; ritt@duke.edu; 4 College of Fisheries and Life Science, Shanghai Ocean University, 999 Hucheng Huan Road, Shanghai 201306, China; lhpeng@shou.edu.cn (L.P.); jlyang@shou.edu.cn (J.-L.Y.)

**Keywords:** functional microbiome, larval settlement, biofouling, marine biofilms, ecosystem services, antifouling, metagenomics, chemical cues, quorum sensing

## Abstract

Natural and artificial marine surfaces are rapidly colonized by microscopic communities, including propagules of some macrofoulers, in a process called biofouling. These microbiomes play an important role in modulating the evolving microbial community, as well as the attachment and settlement of other invertebrate larvae. Microbiomes act as biochemical and biophysical interfaces in marine communities. This review explores the gene-level processes that underlie microbial functions relevant to biofouling and larval settlement, such as quorum sensing, extracellular polymeric substance (EPS), and innate immune system components, as well as biosynthetic and degradative processes that generate signaling molecules. We critically evaluate current knowledge on how microbial metabolites promote or inhibit larval recruitment in corals, barnacles, polychaetes, and bivalves, and how omics-based approaches are uncovering the functional potential of biofilm communities. We evaluate how these interactions influence ecosystem services, such as habitat structuring, reef resilience, and coastal infrastructure maintenance.

## 1. Introduction

**Figure 1:**
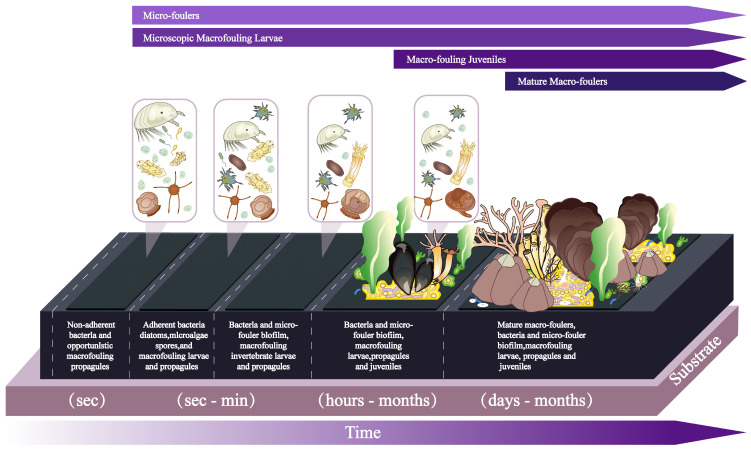
Biofouling process on clean substrata in Beaufort, NC, USA, during warm season.

There are no clean substrates and surfaces in aquatic environments. All substrates in the marine environment are colonized with micro- (bacteria, diatoms, and Protista; later microfouling) and macro-organisms (larvae of invertebrates and spores of algae; later macrofouling). This process is called biofouling [1]. The speed of biofouling and the composition of the communities depend on the geographic conditions, physical and chemical properties of the aquatic environment, and the presence of biofouling organisms [2]. Figure 1 illustrates what happens to surfaces submerged in high salinity water in a temperate environment on the southeast coast of the United States. Variations of this process occur in different environments. In this review, we will focus on the marine environment and marine biofouling.

Larval settlement is a critical phase in the life cycle of many marine invertebrates, including corals, mussels, barnacles, and other attached organisms. This phase determines the successful transition of organisms from planktonic to benthic life. In most cases, larvae settle in places suitable for their future metamorphosis into adults and subsequent growth [3,4,5]. This process is influenced by a variety of environmental cues, among which biofilms play a central role. Biofilms are composed of many species of bacteria and other microorganisms incorporated into a matrix of exopolymer substances [6,7]. These surface-associated microbial communities can produce chemical signals that may induce or inhibit larval attachment, or, in some cases, have no effect [3,5,8]. The composition, structure, and metabolic activity, as well as exudates of microbes in biofilms, are key factors in shaping larval settlement, influencing the distribution and population dynamics of invertebrate species in marine ecosystems. Understanding interactions between biofilms and invertebrate larvae is essential for managing biofouling, designing antifouling solutions, and supporting marine habitat restoration.

Functional microbiome refers to the metabolic activities and functions of microbial communities composed of bacteria, fungi, diatoms, and protists [9], and hosts if they exist. In marine environments, these microbiomes are essential drivers of different ecosystem services, including carbon and biopolymer recycling, nutrient cycling, carbon sequestration, primary production, and the facilitation of larval settlement for habitat-forming species [10,11,12]. Community-forming species like hard corals and oysters indicate their existence with chemicals that enable other members of all life stages to join. Functional microbiomes play a critical role in marine biofouling. Marine microbes change the chemical and physical properties of submerged surfaces through the production of extracellular polymeric substances (EPS), signaling molecules, secondary metabolites, and degradative enzymes [13].

Bacteria within biofilms produce, detect, and respond to chemical signaling molecules in a process that includes quorum sensing (QS) [14,15]. QS can regulate bacterial attachment, motility, biofilm formation, and production of toxins and other chemical compounds. Microbial and host metabolites (e.g., peptides and lipids) can serve as specific chemical cues for larval settlement of macrofouling organisms [13,16]. On the contrary, some bacteria and diatoms can repel larvae and inhibit larval settlement [6,8,17]. Thus, understanding microbial functions in biofilms is crucial for managing biofouling. This knowledge opens the door to environmentally friendly antifouling strategies that target or manipulate microbiome functions rather than killing micro- and macrofouling organisms.

Several recent reviews about the induction of larval settlement by bacteria have been published [3,17,18]. Other publications reviewed antifouling compounds from marine organisms [19,20], marine biofilms [7,21], and quorum sensing and its inhibition in the marine environment [14,22,23,24]. Molecular mechanisms of larval settlement and inhibition were recently reviewed [3,17,25,26]. However, the functional role of marine microbial biofilms in the regulation of biofouling and ecosystem services was not addressed.

The main aim of this review is to synthesize current knowledge on the functional roles of marine microbial biofilms in regulating larval settlement and biofouling dynamics. A particular focus will be the gene-level mechanisms, such as quorum sensing (QS), protein, lipid, and secondary metabolite production and synthesis. Additionally, the role of functional microbiomes in ecosystem services, fouling management, and habitat restoration is assessed.

## 2. Analysis of Publications on Biofouling and Functional Microbiome

**Figure 2:**
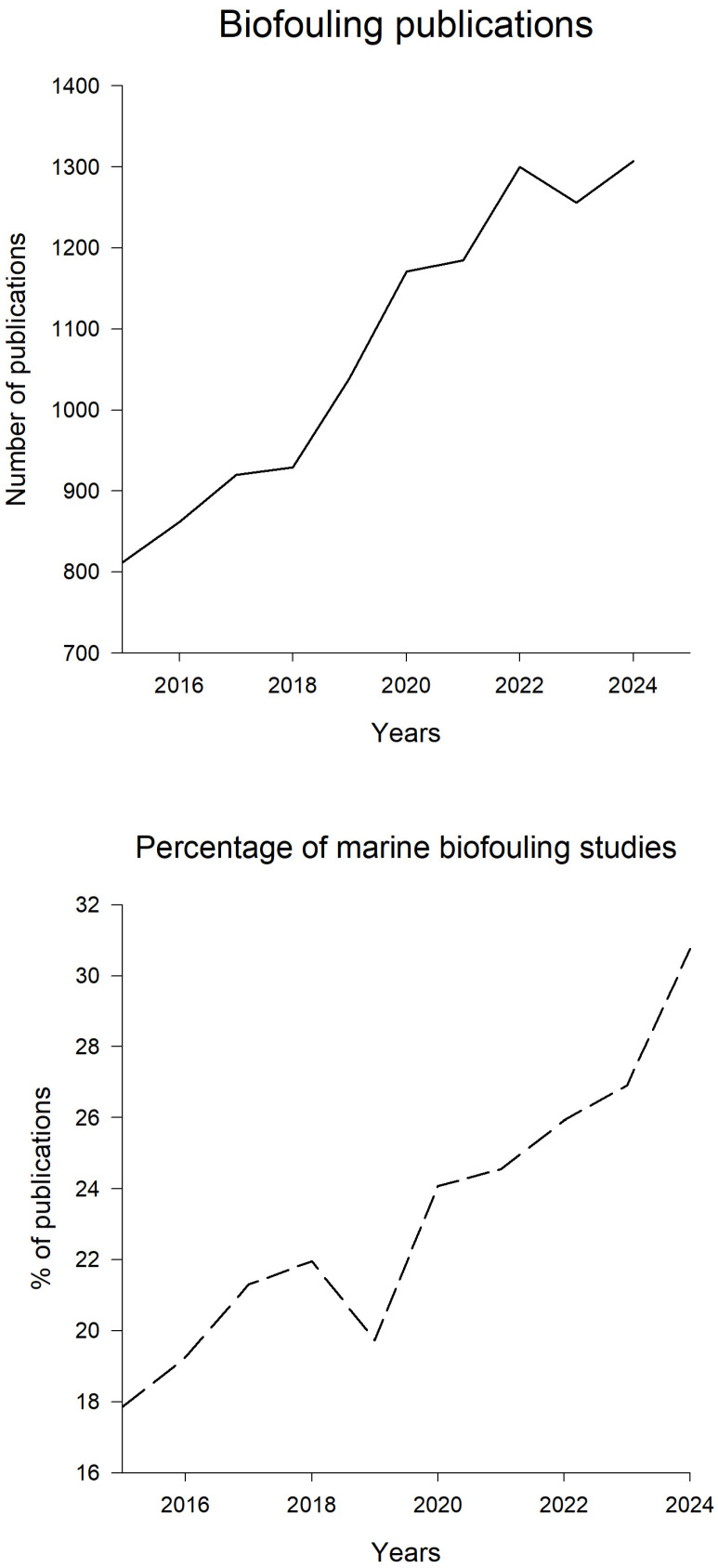
The number of biofouling publications and the percentage of marine biofouling publications for the last 10 years. The search was performed using the SCOPUS database for the period of 2015–2024. The search keywords were “biofouling” for the upper graph, and “biofouling” and “marine” for the lower graph. The data were accessed on 21 August 2025.

Analysis of publications suggested that the number of biofouling papers is increasing over time (Figure 2). In 2024, more than 1300 articles were published in this field. Similarly, the percentage of marine biofouling studies increased over time (Figure 2). About 30% of all publications are on marine biofouling. The rest are medical, industrial, or freshwater biofouling research. Interestingly, the COVID-19 epidemic (2020–2023) did not have any effect on the number of published papers. There was a sharp decline in 2019 in the percentage of marine biofouling publications (Figure 2). There is no clear reason for such a decline in publications, especially due to the fact that the overall number of biofouling studies in that year remains high. Most of the biofouling-related studies focused on species with high economic significance, either harmful, such as bacteria, diatoms, barnacles, and polychaetes, or beneficial, like seaweeds, mussels, and corals [3].

**Figure 3:**
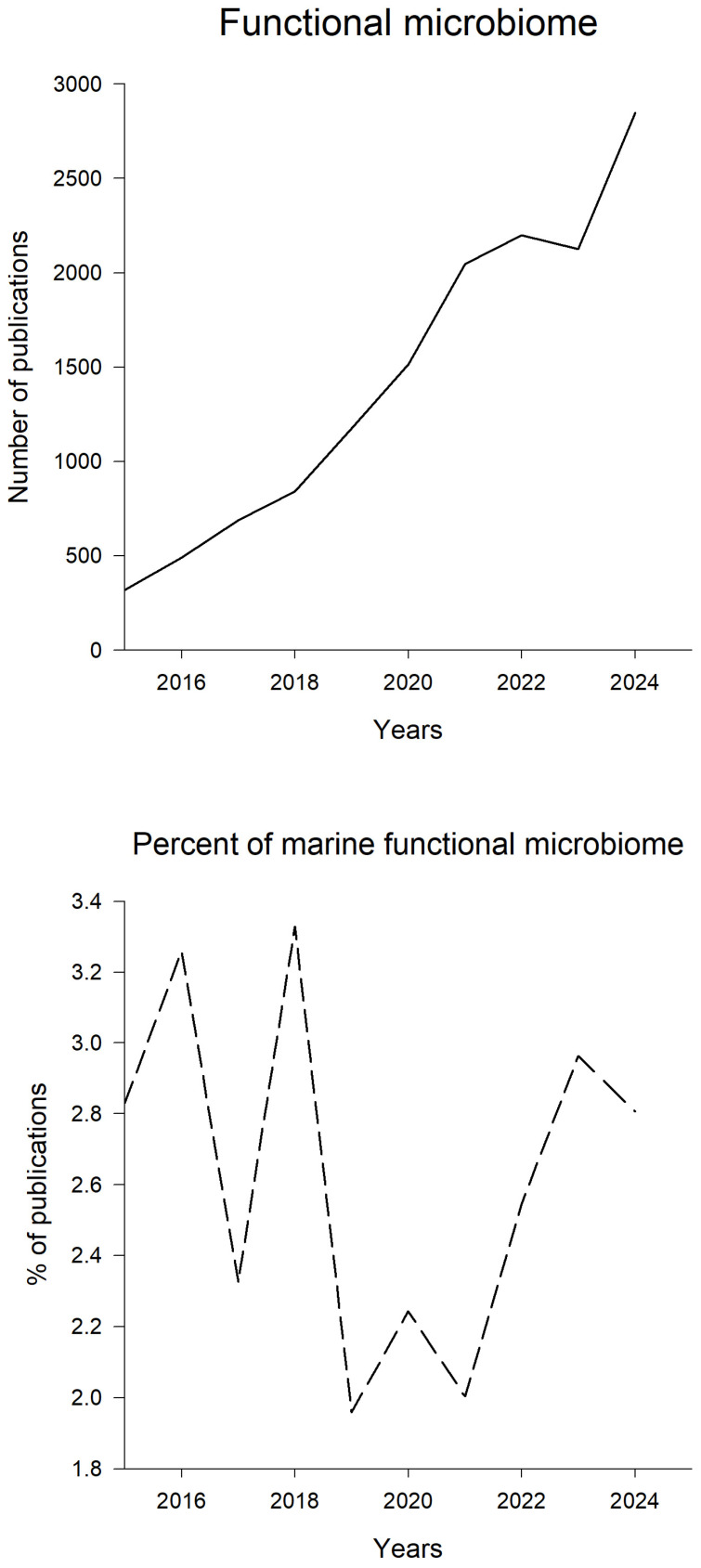
The number of functional microbiome publications and the percentage of marine functional microbiome publications for the last 10 years. The search was performed using the SCOPUS database for the period of 2015–2024. The search keywords were “functional microbiome” for the upper graph, and “functional microbiome” and “marine” for the lower graph. The data were accessed on 21 August 2025.

The field of microbiome research has developed quickly over the past years due to an increase in public interest and funding, the development of molecular techniques, such as metagenome sequencing, and lower costs as the use expands [13]. The term microbiome was proposed by Berg et al. 2020 [27]. The functional microbiome concept is relatively new and has developed because of advances in microbiome research. Analysis of publications on functional microbiomes suggests that only about 300 articles were published on that topic in 2015 (Figure 3). However, in 2024, the number of publications increased almost 9-fold. On the contrary, the number of marine functional microbiome publications remains very small. The percentage of marine functional microbiome publications ranges from 2 to 3 percent (Figure 3). This clearly indicates that this field is a science frontier. There is no clear trend in the marine functional microbiome publications. The lowest percentage of publications was found in 2019 and 2021, while the highest was observed in 2016 and 2018.

## 3. Microbial Biofilm at a Functional Interface

Any submerged surface in the marine environment is colonized by microbes in a process known as microbial succession [6]. Within minutes, surface-active organic molecules adsorb onto the surface, forming a conditioning film that alters its physicochemical properties and facilitates organismal attachment [28]. Within hours, bacteria and other microorganisms, such as cyanobacteria, diatoms, archaea, fungi, and metazoan larvae will appear [8]. At this stage, a well-developed biofilm is formed (Figure 1). Microbes within the biofilm produce extracellular polymeric substances (EPS), creating a matrix that protects microbes and supports further microbial growth and biofilm maturation [28]. During this microbial succession, the community becomes more taxonomically and functionally diverse and composed of thousands of different species.

The composition and dynamics of microbial biofilms vary significantly across different biogeographic regions. In polar waters, microbial communities are often dominated by psychrophilic bacterial taxa such as *Colwellia*, *Marinomonas*, and *Psychromonas* [29]. In contrast, temperate regions tend to host a more seasonally variable community composed of bacteria belonging to the families Rhodobacteraceae and Flavobacteriaceae [30,31]. In contrast, tropical marine environments are characterized by relatively stable environmental conditions and support diverse microbial assemblages, including cyanobacteria, bacteria, and diatoms [7,26,32]. Independent of the community, biological functions are central in providing ecosystem services.

Microbial communities within marine biofilms perform critical ecosystem functions, including heterotrophic processes, biopolymer and nutrient cycling, carbon sequestration, and primary production [7,32]. The most abundant photosynthetic microorganisms in marine biofilms are cyanobacteria and diatoms [33]. They are key primary producers, fixing carbon dioxide through photosynthesis, and are producing organic matter.

Heterotrophic bacteria, including members of the Roseobacter clade, play essential roles in marine biofilms, including anoxygenic chemosynthesis, degradation of organic matter, recycling organic phosphorus, and synthesis of biologically active metabolites [34,35]. Specialized microbes facilitate nitrogen cycling, including nitrogen-fixing bacteria, nitrifying bacteria, denitrifying bacteria, and ammonia-oxidizing archaea and ammonia-oxidizing bacteria [36]. In sulfur cycling, sulfate-reducing bacteria and sulfur-oxidizing bacteria contribute to organic matter degradation and energy flow within biofilms [37].

Symbiotic bacteria and algae play essential roles in the health, physiology, and resilience of marine organisms such as corals, sponges, and seaweeds [38,39]. Symbiotic microbes and host organisms form complex holobiomes that drive nutrient cycling, defense, environmental adaptation, and community structure. For example, researchers identified that putative functions of symbiotic bacteria of the coral *Pocillopora damicornis* include nitrification and denitrification, production of dimethylsulfoniopropionate (DMSP), and supply corals with carbon, amino acids, and vitamins [40]. Well-known symbionts of corals are dinoflagellates of the genus *Symbiodinium* (family Symbiodiniaceae) that live within coral tissues and conduct photosynthesis, supplying the coral with up to 90% of its energy requirements [41]. In return, the algae benefit from a stable coral environment and access to iron, nitrogen, and phosphorus from coral waste. In addition to algae, corals host diverse bacterial communities that perform vital functions such as nitrogen fixation, sulfur cycling, and pathogen defense. For example, the genus *Endozoicomonas* is a dominant bacterial symbiont in many reef-building corals and is thought to aid in nutrient processing and immune modulation [42]. It is present mostly in healthy corals and at lower abundances in corals with abnormal growth [43]. This demonstrates the importance of these bacteria. Some bacteria also produce antimicrobial compounds that inhibit the growth of pathogens [44]. Thus, symbiotic algae and bacteria are fundamental to the ecological function and survival of marine organisms.

Heterotrophic bacteria within microbiomes have a major role in recycling biopolymers, driving the breakdown and reuse of organic matter in ecosystems [45]. This process involves continuous interaction between organisms that produce biopolymers (e.g., for bodily integrity, structural support, cell adhesion, or extracellular matrices) and microbial degraders that utilize these polymers as nutrient sources [46]. Heterotrophs that kill biopolymer producers are called pathogens because they are the basis of diseases that can destroy communities.

To access these energy-rich substrates, heterotrophic microbes secrete specialized degradative enzymes (DNAases, RNAases, lyases, cellulases, proteases, amylases, etc.) that break complex biopolymers into simpler products [47]. These products (e.g., nucleotides, peptides, sugars, amino acids, fatty acids, and simple compounds, like acetic acid, acetate, etc.) can then be assimilated by microbial cells, where they can be used in synthetic pathways or catabolized to generate energy [45]. Additionally, products of degradation act as chemical signatures, generating distinct odors that reflect particular microbial activity and community composition [48]. For individual metazoans, this is species-specific and individual-specific odors, and for communities, this is the odors of reefs that organize associated communities.

Secondary metabolites can be divided into several groups based on their production and the effect on other organisms (Table 1). Common compounds are found in different microbes belonging to different systematic groups. These include small molecules produced through basic fermentation and metabolism, such as acetic acid, dimethyl disulfide, ketones, etc. (Table 1). The seaweed *Ulva fillustrata* produces a defensive metabolite DMSP when the alga is damaged [49]. It is used by seaweed-beneficial bacteria, like *Roseovarius* sp., that stimulate the blade’s growth. *Roseovarius* converted DMPS to dimethyl sulfide (DMS). DMS released by damaged and injured conspecifics stimulates healthy *Ulva* to produce more DMSP. This example demonstrates the complexity of the relationship between bacteria, seaweeds, and their secondary metabolites.

A second group of secondary metabolites includes molecules specifically produced by a particular group of microorganisms (Table 1). Examples of such compounds include geosmin and 2-methylisoborneol, produced only by actinobacteria and some cyanobacteria [50]. While both chemicals are non-toxic, they are used by actinobacteria as warning signals indicating the unpalatability of the strain [51]. In laboratory experiments, geosmin-producing actinobacteria exposed to grazing by the nematode *Caenorhabditis elegans* were stimulated to release toxic metabolites that killed the nematodes. This finding suggests that chemical production triggered by predation can enhance prey defense.

A third group of secondary metabolites includes species-specific compounds produced by one species of bacteria. The macrolide arenicolide produced by marine-derived strains of *Salinispora arenicola* is this type of compound (Table 1). Only bacterial isolates of *S. arenicola* from the Sea of Cortez produce this compound [52]. Arenicolide has potent antimicrobial, antifungal, and anticancer properties [53].

The same compound can have opposite effects on different species. Secondary metabolites like DMS can inhibit the growth of fungi in the biofilm while promoting the growth of some bacteria [54]. DMS produced by bacteria associated with seaweeds and jellyfish could attract sea turtles to areas with high prey density [55]. In conclusion, secondary metabolites link microbial communities to larger biochemical signaling networks, where available resources become organizing information for competitors, hosts, or symbionts.

## 4. Functional Genes Involved in Bacterial EPS Production, Adhesion and Signaling

Functional genes involved in exopolysaccharide (EPS) production of bacteria are essential for survival, adaptation, and ecological interactions in marine environments [56]. EPS are composed mainly of carbohydrates and, to a lesser extent, of proteins [57]. EPS biosynthesis is a complex process regulated by different gene clusters that encode enzymes and transport systems [57]. Genomics reveals that these genes are widely distributed among diverse marine bacterial taxa. In algal-rich habitats, EPS-related bacterial genes are often upregulated and expressed [58]. This suggests their role in biofilm formation and surface colonization. In this study, genes *exoB* and *exoY* were identified as key genetic markers for bacterial EPS production [58]. The gene *exoB* generates UDP-galactose, while *exoY* initiates the synthesis of the primary sugar chain. These findings highlight the importance of EPS gene expression in biofilm development and maturation.

During the initial development of a biofilm, attachment is a two-stage process that begins with a reversible phase, followed by an irreversible one. Weak forces, including electrostatic and hydrophobic interactions, mediate the initial reversible attachment of bacterial cells to the surface. Pili are often involved in the transition between motility and irreversible attachment, for example, the loss of tight adherence (Tad) pili of *Caulobacter crescentus* decreased initial adherence by about 50% [59]. Furthermore, the interaction between pili and the flagellum during the initial contact with the surface initiates a rapid synthesis of holdfast polysaccharide, thereby promoting the transition from reversible to irreversible attachment [60]. Apart from attachment pili, nonfimbrial adhesins are prevalent in bacteria and are important in cell attachment to surfaces. The outer membrane proteins (OMPs) and the outer membrane vesicles (OMVs) of Gram-negative bacteria serve as the primary structural components of the outer membrane and are crucial in regulating bacterial adhesion to surfaces [61,62]. When the outer membrane protein gene *ompX* is inactivated, the interaction between type 1 fimbriae of *Escherichia coli* and non-biological surfaces is enhanced. However, the expression level of OmpX in attached bacteria is significantly lower than that observed in planktonic cells [61,62]. The large adhesion protein, LapA, is associated with the irreversible attachment of *Pseudomonas fluorescens.* Mutants lacking lap exhibit impairments in an early stage of biofilm formation and consequently develop a less mature biofilm [63]. The secretion of LapA is positively regulated by c-di-GMP. The phosphodiesterase RapA inhibits LapA secretion by degrading c-di-GMP [64]. The role of bacterial OMVs and c-di-GMP in larval settlement is discussed in Section 6.

**Figure 4:**
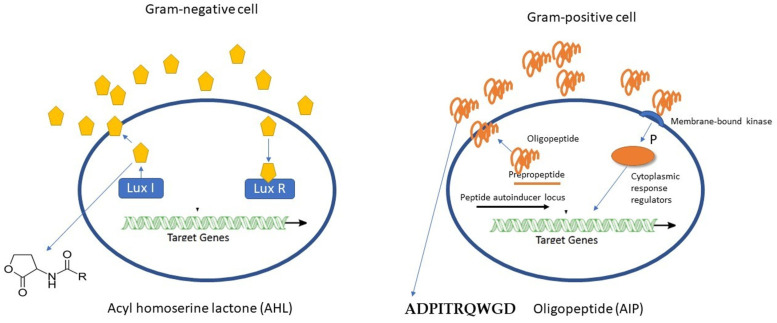
Quorum sensing in Gram-negative and Gram-positive bacteria. P is fatty acid side chain with number of carbons from 4 to 18.

Quorum sensing (QS) is a chemical communication system of bacteria, and it plays a crucial role in the marine environment [14]. In QS, bacteria release and detect small signal molecules (autoinducers) that allow bacteria to coordinate gene expression in a population-density-dependent manner. QS plays a critical role in regulating microbial surface attachment and biofilm development. In Gram-negative bacteria, N-acyl homoserine lactones (AHLs) are common autoinducers [23] (Figure 4). AHLs are produced by LuxI proteins, and they are secreted by the bacteria. Once AHLs intracellular concentration reaches a threshold, they bind to LuxR-type receptors, activating the transcription of genes involved in processes such as biofilm formation, swarming, proteolytic activity, production of toxins and chemicals. In *Vibrio fischeri*, among its three quorum-sensing systems (AinS/AinR, LuxI/LuxR, and LuxS/LuxPQ), only the AinS/AinR system is specifically involved in the initial stage of surface colonization [65]. In *V. cholerae*, all four known quorum-sensing systems (CqsA/CqsS, LuxS/LuxPQ, CqsR, and VpsS) are involved in regulating surface colonization and biofilm formation [66]. Additionally, AHLs produced by bacteria can be detected and used as settlement signals by spores of *Ulva* and larvae of barnacles, *Balanus improvises* [14,24,67]. Similarly, bacterial AHLs induce the release of zoospores from algae *Ulva linza* and *Gracilaria dura* [24].

Unlike Gram-negative bacteria, Gram-positive bacteria primarily employ autoinducing peptides (AIPs) as their QS molecules (Figure 4). These AIPs are typically secreted via dedicated export systems, such as ABC transporters [24]. Upon reaching threshold concentration in the extracellular environment, AIPs are detected by membrane-bound sensor kinases, often part of a two-component regulatory system composed of a sensor kinase and a response regulator (Figure 4). This binding triggers a phosphorylation cascade, ultimately leading to changes in the expression of target genes [68]. QS in Gram-positive bacteria regulates a diverse array of physiological processes, including the production of virulence factors, biofilm formation, sporulation, and antibiotic synthesis [69,70]. Autoinducer-2 (AI-2) is another signaling molecule that enables interspecies communication among both Gram-negative and Gram-positive bacteria [68].

Environmental factors, such as pH, salinity, nutrients, and temperature, can control the concentration of QS molecules. It is known that AHL signals are not stable at low pH, which leads to hydrolysis and degradation [14]. AHLs are relatively stable at normal pH. Longer chain AHLs (>C6) are more stable than short-chain ones. Low temperatures can stimulate production of AHLs by *Yersinia pseudotuberculosis*, while at a temperature higher than 37 °C, AHLs are hydrolyzed [71]. This suggests that QS using AHL molecules is possible only under stable environmental conditions.

A diverse array of strategies has evolved that inhibit bacterial QS, a process referred to as quorum quenching [15]. One of the well-known examples of inhibition of bacterial QS is the Australian red alga *Delisea pulchra*, which produces halogenated furanones that interfere with AHL signaling by competing with binding to LuxR-type receptors [72]. *Delisea* uses furanones to regulate the density and diversity of bacteria on its surface.

Quorum quenching enzymes primarily target acyl-homoserine lactones (AHLs), the key signaling molecules in Gram-negative bacterial QS, by degrading them through hydrolysis [15]. Lactonases hydrolyze the ester bond in the homoserine lactone ring, opening it to form inactive acyl-homoserine, while acylases cleave the amid bond between the acyl side chain and the lactone moiety, producing homoserine lactone and a free fatty acid [14]. The acylase reaction is typically irreversible, unlike lactonase activity, which can reform active AHLs under acidic conditions. Hydrolases are a broader category encompassing both lactonases (e.g., metallo-β-lactamase superfamily enzymes like AiiA) and acylases (e.g., Ntn-hydrolase family), along with other AHL-degrading enzymes that disrupt QS signaling.

Many marine bacteria engage in quorum quenching through enzymatic degradation of AHLs [67,71]. Thus, many bacteria can deactivate QS molecules. Specific examples include bacterial species from the genera *Salinicola*, *Olleya*, and *Erythrobacter*, which degrade AHLs and inhibit biofilm formation by pathogenic bacteria [73,74]. Furthermore, some marine bacteria and other organisms, such as sponges and seaweeds, produce compounds that act as signal antagonists, competing with native autoinducers for receptor binding or interfering with QS signal production [8,67,75]. These diverse strategies highlight the crucial role of QS inhibition in shaping associated microbial communities and mitigating biofouling.

Bacterial QS signals actively mediate interactions with marine plankton, algae, and invertebrates, influencing processes like growth, settlement, and community dynamics [14]. Planktonic microalgae respond to bacterial AHLs both directly, where signals can inhibit algal growth as algicidal agents, and indirectly by regulating bacterial behaviors like motility or biofilm formation that affect algal health [76]. For instance, QS molecules from associated bacteria modulate the microbial loop, with degradation products like tetramic acids [76]. Compounds such as lumichrome protect phytoplankton from pathogens via LasR-like receptors [24]. QS also drives phycosphere succession during algal blooms, stabilizing microbial networks through chemical signaling [77]. Marine invertebrates can use bacterial QS signals as settlement cues [3]. For example, zoospores of the green alga *Ulva* are attracted to AHL-releasing biofilms, facilitating their attachment and colonization [78]. AHLs can induce the settlement of some barnacle species and the settlement behavior of polychaete larvae [79,80]. Eukaryotes like macroalgae and invertebrates interfere via quorum quenching to shape microbiomes, influencing development, disease resistance, and host–virus dynamics in marine ecosystems [14]. These inter-kingdom interactions highlight the importance of QS signals and quorum quenching compounds as key regulators of such processes.

## 5. Immune Response and Antigens Related to Biofouling

Disease is a major evolutionary force [81]. The innate immune response found in all metazoans is an evolutionarily ancient and rapid response to pathogens. The basis of this response is constitutive antibodies capable of recognizing a wide range of antigens expressed by bacteria, but not metazoans. For instance, bacteria commonly express hundreds of different surface carbohydrates containing antigens, whereas metazoans, including humans, express only about ten distinct types [82]. This broad repertoire of antibodies serves as the first line of defense of metazoans against bacteria, particularly those that are involved in decomposition and nutrient cycling. The interaction between pathogens and metazoans is linked to a game of hide-and-seek: pathogens must be undetectable by the metazoan host long enough to infect it and spread the infection to new individuals [83]. One strategy is the expression of metazoan-like antigens, such as blood group antigens, that allow pathogens to “hide” from the host immune system [82]. In fact, some pathogens have evolved the ability to recognize metazoan antigens and exploit them for host cell entry [84]. Examples include bacteria *Listeria* and *Yersinia*, and HIV, which use surface proteins to target specific host cells.

Given the complexity and ancient evolutionary origin of the innate immune response, some metazoan larvae have evolved the ability to recognize bacterial antigens or their secreted products as environmental cues for settlement and metamorphosis [26]. A striking example is found in the polychaete *Hydroides elegans*, whose larvae are triggered to undergo metamorphosis by bacterial O-antigens (also known as H-antigens) [26,85]. Experiments showed that metamorphosis of *H. elegans* is triggered by lipopolysaccharide (LPS), specifically the polysaccharide O-antigen component, from inductive Gram-negative marine biofilm bacteria *Cellulophaga lytica*. LPS from non-inductive bacteria had no activity on larval metamorphosis and did not express O-antigen [85]. This highlights the specificity of the O-antigen structure in the larval developmental process.

Peptide cues generated by trypsin-like serine proteases have a linear sequence, and the specific R groups on the amino acids comprising the sequence are information-rich, much like a word [86]. These kinds of peptides were hypothesized to have evolved a signaling function in circumstances when the release of peptides was directly related to a “life-or-death” event, where receivers of the information would improve fitness. An example of such cues is barnacle settlement pheromone, which enables settlement stage larvae to settle in the proximity of other barnacles, enabling sexual reproduction by sessile adults [87]. Similarly, predatory snails use barnacle settlement pheromone to locate barnacle prey [86]. Predators tapping into a sex pheromone communication system of prey provides a conundrum because barnacles that do not advertise their presence with a sex pheromone are less likely to reproduce. Since barnacle glue curing is mediated by enzyme cascades and reactive oxygen species (ROS) of the innate immune response [17,88,89] and is a major source of pheromone peptides, these peptides are used as information molecules indicating settlement suitability and prey availability.

## 6. Microbiome Cues and Genes Involved in Larval Settlement

Many planktonic heterotrophic bacteria produce extracellular polymeric substances (EPS) in response to reduced nutrient intake [90]. EPS promotes cell aggregation and enables planktonic bacteria to move from the water column to surfaces. Once attached, bacteria capture nutrients and organic molecules while disposing of metabolic waste using flow and biofilm voids with minimal energy expenditure [17].

Some bacteria can chemo-locate surfaces. Upon attachment, the physicochemical nature of the surface significantly influences bacterial physiology [30]. If the surface comprises biopolymers, bacteria recognize them, secrete degradative enzymes and express specific receptors for the uptake of released organic molecules.

Proteins have been initially identified as key chemical inducers for larval settlement. For example, Neumann demonstrated that the inducer for larval metamorphosis of the scyphozoan *Cassiopea andromeda*, is a protein with a molecular weight of approximately 1000–10,000 Da present in the ultrafiltrates of *Vibrio* sp. [91]. In another study, the settlement cue of oyster larvae was identified as a waterborne protein released by adult oysters with molecular weights between 500 and 1000 Da [12,92].

Sensing of the surface can result in the secretion of enzymes that degrade biopolymers. It is inevitable that some enzyme products escape uptake and are predictable odors of degradation [26,86]. The degradation products diffuse into the surrounding environment, creating a distinct chemical signature [86]. These products can be small molecules, such as biogenic amines (skatol, cadaverine, and putrescine), neurotransmitters and nucleotides, DNA and RNA fragments, fatty acids, complex lipids, and short chains of carbohydrates and peptides. Each surface thus acquires a unique chemical odor signature that reflects both its biochemical composition and enzymatic activity. The best documented peptide signals are arginine and lysine carboxyl-terminal peptides generated by trypsin-like serine proteases [93,94]. Another common signal includes substituted amino-sugar disaccharides generated by lyases [93].

Bolger et al. [95] applied the proteomics approach to study the functional microbiomes of two small crustaceans, egg-bearing barnacles and pea crabs. In these animals, the separation of eggs containing developing embryos results in biofouling and embryo mortality. Previous work demonstrated that supplying three specific enzymes, measurable using enzyme-specific substrates, could enable embryo survival through to hatching in blue crabs [88]. Bolger and colleagues expanded on this concept by analyzing peptides generated by exogenous endoproteinases acting on pure proteins to better understand the endoproteinases of the barnacle and pea crab functional microbiomes [95]. Based on the N-terminal sequences of 10,000 to 12,000 peptides produced, 13 families of endoproteinases were identified across the barnacle and pea crab microbiomes. The proportions of enzymes in the two species were different than those found in filtered seawater. However, the pattern of enzymes and their activity was remarkably similar between the two crustacean species during egg hatching [95]. This suggests that these enzymes are produced constitutively; the degradation of the glue binding the eggs in pea crabs or within the mantle cavity of barnacles is facilitated by non-enzymatic mechanisms, potentially involving reductive cleavage of chemical bonds.

Biofilms developed in seawater consist of complex microbial communities that include bacteria, diatoms, and other microorganisms. These biofilms facilitated the settlement of a wide range of invertebrate larvae, including Porifera, Cnidaria, Polychaeta, Mollusca, Echinodermata, Bryozoa, and Brachiopoda [3,5,26]. To evaluate the effects of monospecific biofilms on larval settlement, bacteria are often isolated from natural biofilms. For example, the bacterial strains *Pseudoalteromonas luteoviolacea* and *Cytophaga lytica* could induce the larvae of the polychaete *Hydroides elegans* to undergo settlement and metamorphosis [5]. At the same time, most bacteria cannot be isolated from biofilms, which makes it impossible to study their individual bioactivity. This problem might be solved by metagenomic and metabolomic approaches [13]. Metagenomics of microbial communities is used to study the composition and bioactivity of multispecies biofilms without isolation of single species. Using this approach, it has been shown that the bacterial composition of biofilms on different substrata and in different environments was different, as well as their effect on the settlement of larvae of different species, such as *Hydroides elegans* [26] and *M. coruscus* [17].

The four open reading frames (ORF) in the DNA of *Pseudomonas luteoviolacea* are essential for its capacity to induce the settlement and metamorphosis of larvae of *H. elegans* [96]. Of these four ORFs, two contain conserved domain TIGR02243, encoding components of phage tail-like structures. It was further demonstrated that *P. luteoviolacea* produces arrays of phage tail-like structures (MACs), which consist of 100 contractile elements with outward-facing baseplates, all interconnected by tail fibers and a dynamic hexagonal network [97]. The MACs trigger cilia loss and activate metamorphosis-associated transcription, leading to morphological changes in larvae of *H. elegans* through p38 and c-Jun N-terminal kinase (JNK) MAPK pathways [98]. The induction of larval metamorphosis appears to be strain-specific, which may be attributed to the limited distribution and structural diversity of MACs involved in this process among marine bacteria.

Extensive research has been conducted to identify a universal mechanism for larval metamorphosis by marine bacteria. This has led to the hypothesis that outer membrane vesicles (OMVs), which are conserved products of Gram-negative marine bacteria, may induce larval settlement. OMVs have induced larval metamorphosis in the polychaete *H. elegans* [99] and the mussel *Mytilus couruscus* [100]. The inducing activity of *Cellulophaga lytica* OMVs on *H. elegans* larvae was reduced only under the treatment of lipase [99]. In contrast, treatment of *Pseudoalteromonas marina* OMVs with trypsin, lysozyme, phospholipase C, and lipase decreased their inducing activity on larvae of *M. couruscus* [100]. Due to their complex chemical composition, it remains unclear how OMVs promote the settlement and metamorphosis of larvae.

**Figure 5:**
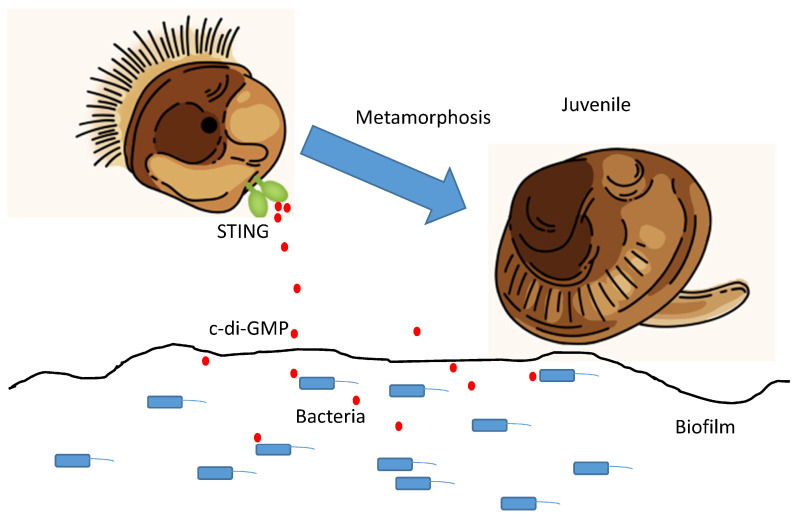
Induction of *Mytilus coruscus* settlement and metamorphosis by the bacterial second messenger c-di-GMP. c-di-GMP produced by the bacterium *P. marina* binds to STING receptor and induces metamorphosis of *M. coruscus* larvae. Adapted from ref. [100].

Bacterial signal molecules (see Section 4) induce larval settlement and metamorphosis. In the bivalve *M. coruscus*, the bacterial second messenger c-di-GMP triggers larval settlement and metamorphosis through binding to the stimulator of interferon genes (STING) receptor [100]. Given its widespread presence in bacteria and its dual regulatory roles in biofilm formation and larval development, c-di-GMP has been proposed as a potential universal signal molecule that induces larval metamorphosis (Figure 5).

Microbial biofilms at functional interfaces not only induce larval settlement but also could prevent it by the production of chitinases. Chitinases, enzymes hydrolyzing β-1,4-linked N-acetylglucosamine in chitin, are integral to certain biofilms [101]. For example, in *Aspergillus fumigatus*, chitinases promote autolysis, leading to extracellular DNA (eDNA) release, enhancing biofilm matrix stability and correlating positively with eDNA secretion [101]. In *Pseudomonas aeruginosa* biofilms, ChiC chitinase is highly abundant in the Pel matrix, contributing to structural integrity and biofilm formation of *P. aeruginosa* despite the bacterium’s inability to catabolize chitin [102]. Beyond biofilm roles, microbial chitinases degrade chitin in barnacle adhesives [103]. It was demonstrated that chitinase compromised attachment and prevented the settlement of *Amphibalanus amphitrite* larvae [103]. These examples suggest that chitinases in biofilms not only support biofilm structure and stability but also impact larval settlement.

Conventional approaches for linking microbial genes to settlement activity typically involve isolating bacterial strains from natural environments and subsequently assessing their effects on larval settlement. Subsequently, functional analysis of microbial genes can be performed through gene editing, where the roles of specific genes are revealed by assessing the impact of gene knockouts on larval settlement and metamorphosis. Liang et al. demonstrated that knockout of the flagellin synthesis gene *fliP* of the bacterium *Pseudomonas marina* significantly impaired the biofilm’s capacity to induce metamorphosis in *M. coruscus* larvae [104]. All four *fliC* genes encoding the subunits of flagellin protein FliC are critical for the induction activity of *P. marina*, as mutations in any gene diminished function. Furthermore, all four recombinant FliC proteins successfully induced metamorphosis in *M. coruscus* larvae, suggesting that the protein’s structural conformation plays a significant role in its inductive effect [105].

Transcriptomics has been used to investigate genes expressed during larval metamorphosis and genes transcribed in biofilms [13]. For example, gene expression profiling across different larval stages reveals that the catecholamine biosynthesis pathway and adrenergic signaling in cardiomyocytes are involved in regulating metamorphosis of *M. coruscus* [106]. A study using a transcriptomic approach showed that the Hedgehog (Hh) signaling pathway and the TGF-beta (TGF-β) signaling pathway are associated with the growth and development of *M. coruscus* larvae [107]. A key challenge remains in linking microbial genes to settlement activity. While single-omics approaches can indicate correlations, integrated multi-omics analyses are necessary to uncover the underlying causal mechanisms.

## 7. Concluding Remarks: Functional Microbiome and Ecological Services

Gene functions and metabolic outputs of microbes can be translated into ecological services. Microbes and hosts can produce enzymes that cleave or modify chemical compounds existing in the biofilm matrix or produced by other organisms. The digestion of a biopolymer generates predictable signature peptides. These signature peptides can be used as signals [86]. Keystone enzymes are exogenous enzymes that generate signals, fragments of existing biopolymer substrates, which are often structural proteins, glycoproteins, and adhesives (reviewed in Section 5). It is no surprise that the evolutionary oldest degradative enzymes produce products that have evolved signaling functions. These signals could be the odor signatures of keystone species, such as corals, oysters, etc. It is not surprising that biochemical signals propagate beyond microbes and influence multicellular organisms. Microbial signals support colonization by conspecifics, symbionts, predators, and other members of the communities [86].

The microbial community and extracellular polymeric substances of biofilms vary with substrate type, which consequently influences their potential to induce larval recruitment [3,26]. Microbial biofilms forming on artificial reef substrates release metabolites that function as chemical cues for larval settlement. Exploiting these biofilm-derived signals represents a promising strategy to enhance recruitment and support restoration of endangered ecosystems, such as coral reefs. This is based on previous studies that show biofilms influenced coral recruitment on different substrata [108]. Additionally, biofilms and their metabolites could serve as effective bio-indicators for rapidly assessing the quality of artificial reef substrates [109] and could be used in the restoration of damaged habitats [110] or prevention of larval settlement (antifouling).

Modern garbage dumps and marine energy extraction infrastructure (oil, gas, and wind) illustrate the complex role of functional microbiomes in organizing biological communities in emerging ecosystems [111]. Garbage dumps contain mixtures of high-energy small organic molecules, partially degraded and intact biopolymers, synthetic polymers, and numerous anthropogenic carcinogens, teratogens, endocrine disruptors, and toxins. These sites are connected to marine environments through air, water, and mobile organisms across multiple trophic levels, demonstrating how communities spontaneously assemble around concentrated energy sources. Although such assemblages are novel and characteristic of the Anthropocene, their organization still reflects fundamental evolutionary relationships among trophic levels and the central role of microbial metabolic functions and products in structuring communities. In contrast to terrestrial garbage dumps, which function as “full-service ecosystems” by providing both energy and structural habitat, marine garbage dumps primarily introduce physical structure into marine environments. This added structure promotes biofilm formation and biofouling communities that efficiently exploit available nutrients and recycle waste. These examples illustrate how microbial functional activity—through the metabolic transformation of energy sources and production of chemical cues—can drive the self-organization of marine communities and shape the development of novel anthropogenic ecosystems.

Although the role of microbiomes in regulating larval settlement is widely recognized, the mechanistic links between specific microbial genes and complex behavioral phenotypes in marine organisms remain poorly understood. Progress is limited by the lack of integrated multi-omics approaches that simultaneously examine microbial biofilm diversity, metabolomes, and proteomes together with host transcriptomic and metabolic responses during settlement [13]. Without coupling biofilm omics with host omics, establishing causal relationships between microbial gene functions and animal behavior remains challenging. Future studies will require integrating multi-omics approaches with controlled experimental models to clarify how functional microbiomes shape the structure and dynamics of marine communities.

## Figures and Tables

**Table 1:** Examples of common, group, and specific metabolic products produced by microbes during biopolymer degradation that can be used as info-chemicals.

Common	Group	Specific
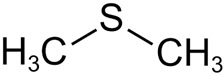 Dimethyl sulfide	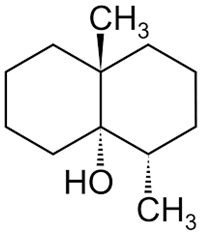 Geosmin	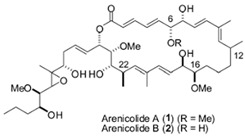 Arenicolide
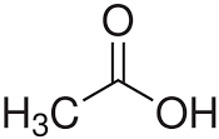 Acetic acid	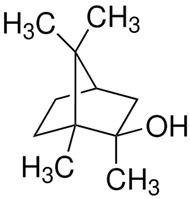 2-methylisoborneol	

## Data Availability

Data can be provided upon request.
